# Bis(1,10-phenanthroline-κ^2^ *N*,*N*′)[2-(4-sulfonato­anilino)acetato-κ*O*]copper(II) dihydrate

**DOI:** 10.1107/S160053681100746X

**Published:** 2011-03-09

**Authors:** Yue Lu, Xing Li, Yue Bing, Mei-Qin Zha, Yin-Xin Li

**Affiliations:** a Faculty of Materials Science and Chemical Engineering, Ningbo University, Ningbo, Zhejiang 315211, People’s Republic of China

## Abstract

In the title compound, [Cu(C_8_H_7_NO_5_S)(C_12_H_8_N_2_)_2_]·2H_2_O, the Cu^II^ ion is coordinated by four N atoms from two 1,10-phenanthroline (phen) ligands and one O atom from a 2-(4-sulfonato­anilino)acetate (spia) ligand in a distorted square-pyramidal geometry. Inter­molecular N—H⋯O and O—H⋯O hydrogen bonds, as well as π–π inter­actions between phen ligands and between phen and spia ligands [centroid–centroid distances = 3.663 (3), 3.768 (3) and 3.565 (3) Å], result in a three-dimensional supra­molecular structure.

## Related literature

For metal complexes with flexible or semi-rigid ligands, see: Chu *et al.* (2008 ▶); Xu *et al.* (2006*a* ▶,*b* ▶); Yong *et al.* (2004 ▶, 2005 ▶).
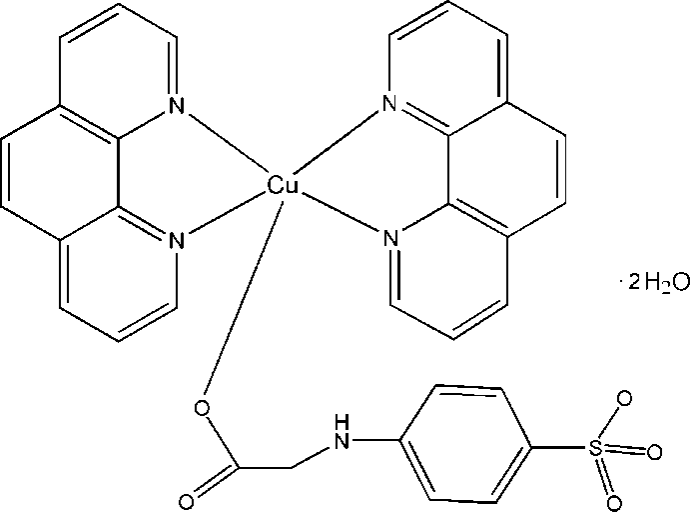


## Experimental

### 

#### Crystal data

[Cu(C_8_H_7_NO_5_S)(C_12_H_8_N_2_)_2_]·2H_2_O
*M* *_r_* = 689.19
Triclinic, 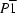

*a* = 9.3437 (19) Å
*b* = 13.274 (3) Å
*c* = 13.880 (3) Å
α = 64.61 (3)°
β = 88.77 (3)°
γ = 69.83 (3)°
*V* = 1443.6 (8) Å^3^
*Z* = 2
Mo *K*α radiation
μ = 0.89 mm^−1^
*T* = 293 K
0.24 × 0.18 × 0.08 mm

#### Data collection

Rigaku R-AXIS RAPID diffractometer
Absorption correction: multi-scan (*ABSCOR*; Higashi, 1995 ▶) *T*_min_ = 0.825, *T*_max_ = 0.931
14102 measured reflections
6590 independent reflections
5238 reflections with *I* > 2σ(*I*)
*R*_int_ = 0.042

#### Refinement

*R*[*F* ^2^ > 2σ(*F* ^2^)] = 0.045
*wR*(*F* ^2^) = 0.130
*S* = 1.11
6590 reflections
409 parameters
H-atom parameters constrained
Δρ_max_ = 0.83 e Å^−3^
Δρ_min_ = −1.99 e Å^−3^

### 

Data collection: *RAPID-AUTO* (Rigaku, 1998 ▶); cell refinement: *RAPID-AUTO*; data reduction: *CrystalStructure* (Rigaku/MSC, 2002 ▶); program(s) used to solve structure: *SHELXS97* (Sheldrick, 2008 ▶); program(s) used to refine structure: *SHELXL97* (Sheldrick, 2008 ▶); molecular graphics: *DIAMOND* (Brandenburg, 1999 ▶); software used to prepare material for publication: *SHELXTL* (Sheldrick, 2008 ▶).

## Supplementary Material

Crystal structure: contains datablocks I, global. DOI: 10.1107/S160053681100746X/hy2409sup1.cif

Structure factors: contains datablocks I. DOI: 10.1107/S160053681100746X/hy2409Isup2.hkl

Additional supplementary materials:  crystallographic information; 3D view; checkCIF report

## Figures and Tables

**Table 1:** Selected bond lengths (Å)

Cu1—O2	1.993 (2)
Cu1—N1	1.999 (2)
Cu1—N2	2.049 (2)
Cu1—N3	2.005 (2)
Cu1—N4	2.212 (3)

**Table 2:** Hydrogen-bond geometry (Å, °)

*D*—H⋯*A*	*D*—H	H⋯*A*	*D*⋯*A*	*D*—H⋯*A*
N5—H5*A*⋯O2^i^	0.86	2.35	3.173 (4)	160
O6—H6*A*⋯O4	0.76	2.11	2.850 (5)	166
O6—H6*B*⋯O1^ii^	0.86	2.15	2.963 (5)	156
O7—H7*B*⋯O3^iii^	0.72	2.24	2.915 (4)	156
O7—H7*A*⋯O5^iv^	0.76	2.11	2.785 (4)	148

Symmetry codes: (i) 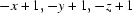; (ii) 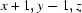; (iii) 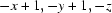; (iv) 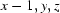.

**Fig. 1.**
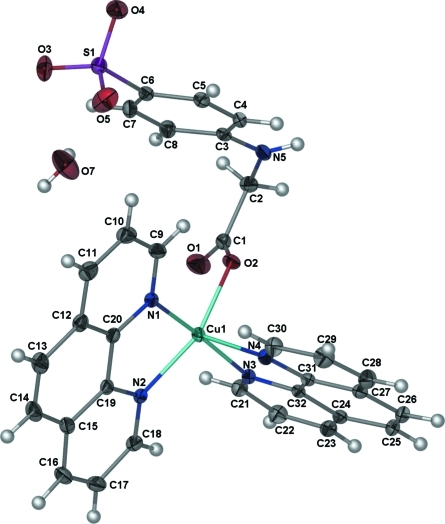
Molecular structure of the title compound. Displacement ellipsoids are drawn at the 30% probability level.

**Fig. 2.**
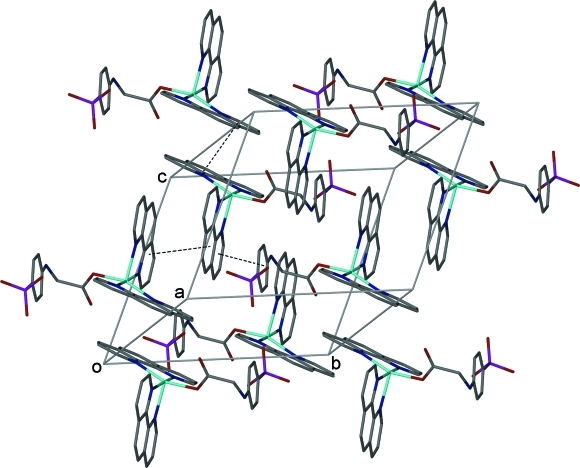
Crystal packing of the title compound, showing π–π stacking interactions (dashed lines) between the phen ligands [centroid–centroid distances = 3.663 (3) and 3.768 (3) Å] and between the phen and spia ligands [centroid–centroid distance = 3.565 (3) Å].

## supplementary crystallographic information

### Comment

Flexible or semi-rigid ligands can adopt various conformations and coordination modes according to the geometric requirements of different metal ions, which have attracted more attention in the fields of supramolecular chemistry (Chu *et al.*, 2008; Xu *et al.*, 2006*a*,b; Yong *et al.*, 2004, 2005). Here we use N-(4-sulfanilicphenyl)iminoacetic acid (H_2_spia) and CuSO_4_.5H_2_O to prepare a copper compound with the spia ligand. The title compound is a mononuclear complex, with five-coordinated Cu^II^ ions. As shown in Fig. 1, the Cu^II^ ion is coordinated by one O atom from an spia ligand and four N atoms from two 1,10-phenanthroline ligands. There are two uncoordinated water molecules in the asymmetric unit.

### Experimental

H_2_spia was prepared following the method described by Yong *et al.* (2005). A solution of KOH (2.694 g, 48 mmol) in water (5 ml) was added dropwise to chloroacetic acid sodium salt (2.796 g, 24 mmol) in water (5 ml) with stirring. Sulfanilic acid (1.044 g, 6 mmol) was slowly added to the reaction mixture and KI (0.025 g) was added as catalyst. Then the mixture was refluxed at about 80°C for 30 h. The reaction solution was cooled to room temperature and acidified with HCl (6 mol/*L*) until the desired white acidic material precipitated (pH = 3), which was filtered, washed with water and dried in air.

The title compound was prepared by a solvent evaporation method. A mixture of CuSO_4_.5H_2_O (0.025 g, 0.1 mmol), H_2_spia (0.029 g, 0.1 mmol), and 1,10-phenanthroline (0.040 g, 0.20 mmol) in 15 ml of water was heated for 30 min. One drop of KOH solution was added to adjust pH to 5, and then the mixture was filtered. Dark green single crystals suitable for X-ray analysis were obtained by slow evaporation of solvent at room temperature.

### Refinement

H atoms attached to C and N atoms were positioned geometrically and refined as riding atoms, with C—H = 0.93 (CH) and 0.98 (CH_2_), N—H = 0.86 Å and with *U*_iso_(H) = 1.2*U*_eq_(C, N). H atoms of water molecules were found in a difference Fourier map and refined as riding atoms, with *U*_iso_(H) = 1.2*U*_eq_(O).

### Crystal data

**Table:**

[Cu(C_8_H_7_NO_5_S)(C_12_H_8_N_2_)_2_]·2H_2_O	*Z* = 2
*M**_r_* = 689.19	*F*(000) = 710
Triclinic, *P*1	*D*_x_ = 1.585 Mg m^−^^3^
Hall symbol: -P 1	Mo *K*α radiation, λ = 0.71073 Å
*a* = 9.3437 (19) Å	Cell parameters from 14102 reflections
*b* = 13.274 (3) Å	θ = 3.1–27.5°
*c* = 13.880 (3) Å	µ = 0.89 mm^−^^1^
α = 64.61 (3)°	*T* = 293 K
β = 88.77 (3)°	Platelet, dark green
γ = 69.83 (3)°	0.24 × 0.18 × 0.08 mm
*V* = 1443.6 (8) Å^3^	

### Data collection

**Table:**

Rigaku R-AXIS RAPID diffractometer	6590 independent reflections
Radiation source: rotation anode	5238 reflections with *I* > 2σ(*I*)
graphite	*R*_int_ = 0.042
ω scans	θ_max_ = 27.5°, θ_min_ = 3.1°
Absorption correction: multi-scan (*ABSCOR*; Higashi, 1995)	*h* = −11→12
*T*_min_ = 0.825, *T*_max_ = 0.931	*k* = −17→17
14102 measured reflections	*l* = −17→17

### Refinement

**Table:**

Refinement on *F*^2^	Primary atom site location: structure-invariant direct methods
Least-squares matrix: full	Secondary atom site location: difference Fourier map
*R*[*F*^2^ > 2σ(*F*^2^)] = 0.045	Hydrogen site location: inferred from neighbouring sites
*wR*(*F*^2^) = 0.130	H-atom parameters constrained
*S* = 1.11	*w* = 1/[σ^2^(*F*_o_^2^) + (0.0596*P*)^2^ + 1.1815*P*] where *P* = (*F*_o_^2^ + 2*F*_c_^2^)/3
6590 reflections	(Δ/σ)_max_ = 0.001
409 parameters	Δρ_max_ = 0.83 e Å^−^^3^
0 restraints	Δρ_min_ = −1.98 e Å^−^^3^

### Fractional atomic coordinates and isotropic or equivalent isotropic displacement parameters (Å^2^)

**Table:**

	*x*	*y*	*z*	*U*_iso_*/*U*_eq_	
Cu1	0.50512 (4)	0.83070 (3)	0.27467 (2)	0.02821 (11)	
S1	0.82946 (9)	0.30436 (7)	0.13978 (6)	0.03859 (18)	
O1	0.2037 (3)	0.7912 (2)	0.2589 (2)	0.0650 (7)	
O2	0.4453 (2)	0.68782 (18)	0.34176 (16)	0.0363 (5)	
O3	0.7572 (3)	0.3723 (2)	0.02907 (19)	0.0601 (7)	
O4	0.8555 (2)	0.17807 (19)	0.18310 (17)	0.03859 (18)	
O5	0.9698 (3)	0.3209 (3)	0.1589 (3)	0.0700 (8)	
O6	0.8946 (4)	−0.0692 (3)	0.2861 (3)	0.0805 (9)	
H6B	0.9681	−0.1096	0.2628	0.097*	
H6A	0.8849	−0.0049	0.2496	0.097*	
O7	0.2154 (3)	0.3957 (3)	0.0958 (3)	0.0848 (10)	
H7B	0.2070	0.4536	0.0521	0.102*	
H7A	0.1317	0.4014	0.0956	0.102*	
N1	0.6589 (3)	0.7539 (2)	0.20033 (18)	0.0299 (5)	
N2	0.5263 (3)	0.9827 (2)	0.15768 (17)	0.0286 (5)	
N3	0.3742 (3)	0.9116 (2)	0.35647 (17)	0.0280 (5)	
N4	0.6770 (3)	0.7688 (2)	0.41483 (18)	0.0307 (5)	
N5	0.4049 (3)	0.4718 (2)	0.4122 (2)	0.0372 (6)	
H5A	0.4238	0.4250	0.4803	0.045*	
C1	0.3090 (4)	0.6979 (3)	0.3178 (2)	0.0355 (6)	
C2	0.2737 (3)	0.5822 (3)	0.3707 (3)	0.0379 (7)	
H2A	0.2164	0.5843	0.4294	0.046*	
H2B	0.2073	0.5823	0.3179	0.046*	
C3	0.5021 (3)	0.4384 (2)	0.3459 (2)	0.0322 (6)	
C4	0.6514 (3)	0.3536 (2)	0.3900 (2)	0.0339 (6)	
H4	0.6851	0.3226	0.4631	0.041*	
C5	0.7493 (3)	0.3154 (3)	0.3265 (2)	0.0342 (6)	
H6	0.8485	0.2593	0.3573	0.041*	
C6	0.7018 (3)	0.3596 (2)	0.2172 (2)	0.0321 (6)	
C7	0.5545 (3)	0.4453 (3)	0.1722 (2)	0.0367 (6)	
H7	0.5219	0.4765	0.0989	0.044*	
C8	0.4560 (4)	0.4848 (3)	0.2347 (2)	0.0372 (6)	
H5	0.3581	0.5428	0.2031	0.045*	
C9	0.7205 (3)	0.6388 (3)	0.2225 (2)	0.0363 (6)	
H9	0.6801	0.5856	0.2715	0.044*	
C10	0.8439 (4)	0.5948 (3)	0.1746 (3)	0.0442 (7)	
H10	0.8843	0.5136	0.1916	0.053*	
C11	0.9054 (4)	0.6718 (3)	0.1025 (3)	0.0447 (7)	
H11	0.9889	0.6429	0.0713	0.054*	
C12	0.8420 (3)	0.7936 (3)	0.0762 (2)	0.0361 (6)	
C13	0.8923 (4)	0.8835 (3)	−0.0014 (3)	0.0457 (8)	
H13	0.9745	0.8606	−0.0362	0.055*	
C14	0.8231 (4)	0.9996 (3)	−0.0246 (3)	0.0455 (8)	
H14	0.8580	1.0555	−0.0757	0.055*	
C15	0.6969 (3)	1.0401 (3)	0.0272 (2)	0.0356 (6)	
C16	0.6218 (4)	1.1597 (3)	0.0083 (3)	0.0451 (8)	
H16	0.6543	1.2192	−0.0397	0.054*	
C17	0.5005 (4)	1.1882 (3)	0.0611 (3)	0.0453 (8)	
H17	0.4483	1.2676	0.0479	0.054*	
C18	0.4555 (4)	1.0975 (3)	0.1349 (2)	0.0375 (6)	
H18	0.3722	1.1184	0.1696	0.045*	
C19	0.6454 (3)	0.9541 (2)	0.1040 (2)	0.0287 (5)	
C20	0.7174 (3)	0.8311 (2)	0.1275 (2)	0.0289 (5)	
C21	0.2280 (3)	0.9850 (3)	0.3261 (2)	0.0347 (6)	
H21	0.1803	1.0065	0.2583	0.042*	
C22	0.1422 (4)	1.0315 (3)	0.3907 (3)	0.0403 (7)	
H22	0.0398	1.0836	0.3660	0.048*	
C23	0.2101 (3)	0.9997 (3)	0.4914 (2)	0.0371 (6)	
H23	0.1542	1.0301	0.5356	0.044*	
C24	0.3645 (3)	0.9212 (2)	0.5271 (2)	0.0295 (6)	
C25	0.4415 (4)	0.8779 (3)	0.6331 (2)	0.0368 (6)	
H25	0.3886	0.9037	0.6808	0.044*	
C26	0.5888 (4)	0.8008 (3)	0.6650 (2)	0.0383 (7)	
H26	0.6350	0.7717	0.7352	0.046*	
C27	0.6758 (3)	0.7627 (2)	0.5921 (2)	0.0322 (6)	
C28	0.8313 (4)	0.6867 (3)	0.6195 (3)	0.0432 (7)	
H28	0.8838	0.6579	0.6878	0.052*	
C29	0.9051 (4)	0.6555 (3)	0.5448 (3)	0.0481 (8)	
H29	1.0088	0.6059	0.5617	0.058*	
C30	0.8241 (3)	0.6985 (3)	0.4431 (3)	0.0399 (7)	
H30	0.8762	0.6766	0.3930	0.048*	
C31	0.6037 (3)	0.8018 (2)	0.4881 (2)	0.0269 (5)	
C32	0.4447 (3)	0.8800 (2)	0.4561 (2)	0.0262 (5)	

### Atomic displacement parameters (Å^2^)

**Table:**

	*U*^11^	*U*^22^	*U*^33^	*U*^12^	*U*^13^	*U*^23^
Cu1	0.03390 (19)	0.02680 (19)	0.02459 (18)	−0.01173 (14)	0.00970 (12)	−0.01201 (13)
S1	0.0437 (4)	0.0380 (4)	0.0373 (4)	−0.0160 (3)	0.0141 (3)	−0.0195 (3)
O1	0.0678 (17)	0.0393 (14)	0.0659 (18)	−0.0111 (13)	−0.0156 (14)	−0.0099 (13)
O2	0.0408 (11)	0.0305 (10)	0.0413 (12)	−0.0188 (9)	0.0159 (9)	−0.0157 (9)
O3	0.0700 (17)	0.0599 (16)	0.0314 (12)	−0.0063 (14)	0.0115 (11)	−0.0178 (11)
O4	0.0437 (4)	0.0380 (4)	0.0373 (4)	−0.0160 (3)	0.0141 (3)	−0.0195 (3)
O5	0.0512 (15)	0.106 (2)	0.092 (2)	−0.0452 (17)	0.0369 (15)	−0.068 (2)
O6	0.073 (2)	0.0683 (19)	0.094 (2)	−0.0366 (17)	0.0195 (17)	−0.0232 (17)
O7	0.0587 (17)	0.072 (2)	0.078 (2)	−0.0235 (16)	−0.0072 (15)	0.0056 (16)
N1	0.0365 (12)	0.0315 (12)	0.0264 (11)	−0.0165 (10)	0.0094 (9)	−0.0144 (9)
N2	0.0342 (12)	0.0281 (11)	0.0240 (11)	−0.0109 (10)	0.0028 (9)	−0.0125 (9)
N3	0.0318 (11)	0.0278 (11)	0.0242 (11)	−0.0112 (10)	0.0050 (9)	−0.0115 (9)
N4	0.0305 (11)	0.0330 (12)	0.0303 (12)	−0.0130 (10)	0.0078 (9)	−0.0148 (10)
N5	0.0506 (15)	0.0279 (12)	0.0327 (13)	−0.0158 (11)	0.0167 (11)	−0.0128 (10)
C1	0.0452 (17)	0.0315 (15)	0.0304 (15)	−0.0134 (14)	0.0094 (12)	−0.0153 (12)
C2	0.0390 (16)	0.0378 (16)	0.0457 (17)	−0.0195 (14)	0.0189 (13)	−0.0229 (14)
C3	0.0439 (16)	0.0270 (13)	0.0319 (14)	−0.0212 (13)	0.0141 (12)	−0.0129 (11)
C4	0.0467 (16)	0.0285 (14)	0.0247 (13)	−0.0155 (13)	0.0052 (11)	−0.0092 (11)
C5	0.0384 (15)	0.0269 (13)	0.0328 (15)	−0.0101 (12)	0.0034 (11)	−0.0108 (11)
C6	0.0391 (15)	0.0299 (14)	0.0318 (14)	−0.0174 (13)	0.0113 (11)	−0.0146 (11)
C7	0.0421 (16)	0.0373 (16)	0.0266 (14)	−0.0131 (14)	0.0049 (11)	−0.0121 (12)
C8	0.0374 (15)	0.0352 (15)	0.0340 (15)	−0.0094 (13)	0.0032 (12)	−0.0140 (12)
C9	0.0401 (16)	0.0310 (15)	0.0410 (16)	−0.0146 (13)	0.0129 (12)	−0.0182 (13)
C10	0.0457 (18)	0.0367 (16)	0.053 (2)	−0.0115 (15)	0.0120 (15)	−0.0261 (15)
C11	0.0402 (17)	0.055 (2)	0.0489 (19)	−0.0174 (16)	0.0181 (14)	−0.0335 (16)
C12	0.0354 (15)	0.0477 (17)	0.0345 (15)	−0.0204 (14)	0.0121 (12)	−0.0230 (13)
C13	0.0421 (17)	0.061 (2)	0.0409 (18)	−0.0271 (17)	0.0189 (14)	−0.0233 (16)
C14	0.0472 (18)	0.056 (2)	0.0363 (17)	−0.0332 (17)	0.0127 (13)	−0.0129 (15)
C15	0.0400 (15)	0.0404 (16)	0.0290 (14)	−0.0238 (14)	0.0016 (11)	−0.0107 (12)
C16	0.0546 (19)	0.0388 (17)	0.0385 (17)	−0.0269 (16)	0.0012 (14)	−0.0068 (13)
C17	0.056 (2)	0.0293 (15)	0.0463 (19)	−0.0153 (15)	−0.0018 (15)	−0.0135 (14)
C18	0.0459 (17)	0.0331 (15)	0.0347 (15)	−0.0128 (13)	0.0038 (12)	−0.0177 (12)
C19	0.0315 (13)	0.0345 (14)	0.0227 (12)	−0.0160 (12)	0.0020 (10)	−0.0121 (11)
C20	0.0319 (13)	0.0354 (14)	0.0252 (13)	−0.0167 (12)	0.0067 (10)	−0.0156 (11)
C21	0.0315 (14)	0.0349 (15)	0.0342 (15)	−0.0074 (12)	0.0024 (11)	−0.0162 (12)
C22	0.0326 (15)	0.0379 (16)	0.0456 (18)	−0.0082 (13)	0.0066 (12)	−0.0185 (14)
C23	0.0405 (16)	0.0360 (15)	0.0405 (16)	−0.0148 (13)	0.0165 (12)	−0.0224 (13)
C24	0.0356 (14)	0.0277 (13)	0.0301 (14)	−0.0161 (12)	0.0112 (11)	−0.0142 (11)
C25	0.0509 (18)	0.0376 (16)	0.0277 (14)	−0.0181 (14)	0.0120 (12)	−0.0186 (12)
C26	0.0523 (18)	0.0357 (15)	0.0265 (14)	−0.0156 (14)	0.0022 (12)	−0.0140 (12)
C27	0.0388 (15)	0.0278 (13)	0.0290 (14)	−0.0135 (12)	0.0012 (11)	−0.0110 (11)
C28	0.0409 (17)	0.0435 (18)	0.0381 (17)	−0.0108 (15)	−0.0076 (13)	−0.0154 (14)
C29	0.0316 (15)	0.051 (2)	0.052 (2)	−0.0060 (15)	−0.0022 (13)	−0.0211 (16)
C30	0.0315 (14)	0.0434 (17)	0.0430 (17)	−0.0100 (13)	0.0089 (12)	−0.0210 (14)
C31	0.0324 (13)	0.0236 (12)	0.0261 (13)	−0.0130 (11)	0.0059 (10)	−0.0105 (10)
C32	0.0321 (13)	0.0235 (12)	0.0245 (12)	−0.0130 (11)	0.0074 (10)	−0.0101 (10)

### Geometric parameters (Å, °)

**Table:**

Cu1—O2	1.993 (2)	C9—H9	0.9300
Cu1—N1	1.999 (2)	C10—C11	1.371 (5)
Cu1—N2	2.049 (2)	C10—H10	0.9300
Cu1—N3	2.005 (2)	C11—C12	1.394 (5)
Cu1—N4	2.212 (3)	C11—H11	0.9300
S1—O3	1.438 (3)	C12—C20	1.406 (4)
S1—O4	1.446 (2)	C12—C13	1.438 (4)
S1—O5	1.450 (3)	C13—C14	1.338 (5)
S1—C6	1.769 (3)	C13—H13	0.9300
O1—C1	1.223 (4)	C14—C15	1.434 (4)
O2—C1	1.270 (4)	C14—H14	0.9300
O6—H6B	0.86	C15—C16	1.401 (5)
O6—H6A	0.76	C15—C19	1.409 (4)
O7—H7B	0.72	C16—C17	1.369 (5)
O7—H7A	0.76	C16—H16	0.9300
N1—C9	1.326 (4)	C17—C18	1.396 (4)
N1—C20	1.359 (3)	C17—H17	0.9300
N2—C18	1.326 (4)	C18—H18	0.9300
N2—C19	1.362 (3)	C19—C20	1.421 (4)
N3—C21	1.322 (4)	C21—C22	1.389 (4)
N3—C32	1.369 (3)	C21—H21	0.9300
N4—C30	1.319 (4)	C22—C23	1.372 (4)
N4—C31	1.356 (3)	C22—H22	0.9300
N5—C3	1.380 (4)	C23—C24	1.401 (4)
N5—C2	1.432 (4)	C23—H23	0.9300
N5—H5A	0.8600	C24—C32	1.407 (4)
C1—C2	1.543 (4)	C24—C25	1.431 (4)
C2—H2A	0.9700	C25—C26	1.343 (4)
C2—H2B	0.9700	C25—H25	0.9300
C3—C4	1.397 (4)	C26—C27	1.435 (4)
C3—C8	1.405 (4)	C26—H26	0.9300
C4—C5	1.377 (4)	C27—C28	1.399 (4)
C4—H4	0.9300	C27—C31	1.402 (4)
C5—C6	1.390 (4)	C28—C29	1.365 (5)
C5—H6	0.9300	C28—H28	0.9300
C6—C7	1.388 (4)	C29—C30	1.397 (5)
C7—C8	1.378 (4)	C29—H29	0.9300
C7—H7	0.9300	C30—H30	0.9300
C8—H5	0.9300	C31—C32	1.432 (4)
C9—C10	1.395 (4)		
			
O2—Cu1—N1	91.43 (9)	C10—C11—C12	119.6 (3)
O2—Cu1—N3	93.36 (9)	C10—C11—H11	120.2
N1—Cu1—N3	172.29 (9)	C12—C11—H11	120.2
O2—Cu1—N2	159.24 (9)	C11—C12—C20	117.3 (3)
N1—Cu1—N2	81.66 (9)	C11—C12—C13	124.6 (3)
N3—Cu1—N2	95.79 (9)	C20—C12—C13	118.0 (3)
O2—Cu1—N4	94.19 (9)	C14—C13—C12	121.3 (3)
N1—Cu1—N4	93.85 (9)	C14—C13—H13	119.3
N3—Cu1—N4	79.78 (9)	C12—C13—H13	119.3
N2—Cu1—N4	105.74 (9)	C13—C14—C15	121.8 (3)
O3—S1—O4	113.06 (15)	C13—C14—H14	119.1
O3—S1—O5	113.39 (18)	C15—C14—H14	119.1
O4—S1—O5	110.79 (17)	C16—C15—C19	117.2 (3)
O3—S1—C6	107.61 (15)	C16—C15—C14	124.6 (3)
O4—S1—C6	106.23 (13)	C19—C15—C14	118.2 (3)
O5—S1—C6	105.13 (15)	C17—C16—C15	119.5 (3)
C1—O2—Cu1	119.80 (19)	C17—C16—H16	120.3
H6B—O6—H6A	102.9	C15—C16—H16	120.3
H7B—O7—H7A	100.4	C16—C17—C18	119.6 (3)
C9—N1—C20	118.4 (2)	C16—C17—H17	120.2
C9—N1—Cu1	128.42 (19)	C18—C17—H17	120.2
C20—N1—Cu1	112.63 (18)	N2—C18—C17	122.9 (3)
C18—N2—C19	117.8 (2)	N2—C18—H18	118.5
C18—N2—Cu1	131.1 (2)	C17—C18—H18	118.5
C19—N2—Cu1	110.75 (18)	N2—C19—C15	123.0 (3)
C21—N3—C32	118.6 (2)	N2—C19—C20	117.0 (2)
C21—N3—Cu1	126.62 (19)	C15—C19—C20	120.0 (3)
C32—N3—Cu1	114.69 (18)	N1—C20—C12	122.8 (3)
C30—N4—C31	117.8 (2)	N1—C20—C19	116.6 (2)
C30—N4—Cu1	132.9 (2)	C12—C20—C19	120.6 (2)
C31—N4—Cu1	108.84 (17)	N3—C21—C22	123.1 (3)
C3—N5—C2	121.9 (2)	N3—C21—H21	118.4
C3—N5—H5A	119.1	C22—C21—H21	118.4
C2—N5—H5A	119.1	C23—C22—C21	119.2 (3)
O1—C1—O2	125.7 (3)	C23—C22—H22	120.4
O1—C1—C2	117.8 (3)	C21—C22—H22	120.4
O2—C1—C2	116.4 (3)	C22—C23—C24	119.5 (3)
N5—C2—C1	115.8 (2)	C22—C23—H23	120.3
N5—C2—H2A	108.3	C24—C23—H23	120.3
C1—C2—H2A	108.3	C23—C24—C32	118.0 (3)
N5—C2—H2B	108.3	C23—C24—C25	123.1 (3)
C1—C2—H2B	108.3	C32—C24—C25	118.9 (3)
H2A—C2—H2B	107.4	C26—C25—C24	121.3 (3)
N5—C3—C4	119.5 (3)	C26—C25—H25	119.3
N5—C3—C8	122.5 (3)	C24—C25—H25	119.3
C4—C3—C8	118.0 (3)	C25—C26—C27	120.8 (3)
C5—C4—C3	120.9 (3)	C25—C26—H26	119.6
C5—C4—H4	119.6	C27—C26—H26	119.6
C3—C4—H4	119.6	C28—C27—C31	117.3 (3)
C4—C5—C6	120.9 (3)	C28—C27—C26	123.2 (3)
C4—C5—H6	119.6	C31—C27—C26	119.5 (3)
C6—C5—H6	119.6	C29—C28—C27	119.2 (3)
C7—C6—C5	118.7 (3)	C29—C28—H28	120.4
C7—C6—S1	122.0 (2)	C27—C28—H28	120.4
C5—C6—S1	119.3 (2)	C28—C29—C30	119.7 (3)
C8—C7—C6	120.9 (3)	C28—C29—H29	120.2
C8—C7—H7	119.6	C30—C29—H29	120.2
C6—C7—H7	119.6	N4—C30—C29	122.8 (3)
C7—C8—C3	120.7 (3)	N4—C30—H30	118.6
C7—C8—H5	119.7	C29—C30—H30	118.6
C3—C8—H5	119.7	N4—C31—C27	123.2 (3)
N1—C9—C10	122.2 (3)	N4—C31—C32	117.4 (2)
N1—C9—H9	118.9	C27—C31—C32	119.4 (2)
C10—C9—H9	118.9	N3—C32—C24	121.6 (2)
C11—C10—C9	119.7 (3)	N3—C32—C31	118.4 (2)
C11—C10—H10	120.1	C24—C32—C31	120.0 (2)
C9—C10—H10	120.1		

### Hydrogen-bond geometry (Å, °)

**Table:**

*D*—H···*A*	*D*—H	H···*A*	*D*···*A*	*D*—H···*A*
N5—H5A···O2^i^	0.86	2.35	3.173 (4)	160
O6—H6A···O4	0.76	2.11	2.850 (5)	166
O6—H6B···O1^ii^	0.86	2.15	2.963 (5)	156
O7—H7B···O3^iii^	0.72	2.24	2.915 (4)	156
O7—H7A···O5^iv^	0.76	2.11	2.785 (4)	148

Symmetry codes: (i) −*x*+1, −*y*+1, −*z*+1; (ii) *x*+1, *y*−1, *z*; (iii) −*x*+1, −*y*+1, −*z*; (iv) *x*−1, *y*, *z*.
